# Prediction of respiratory diseases based on random forest model

**DOI:** 10.3389/fpubh.2025.1537238

**Published:** 2025-02-14

**Authors:** Xiaotong Yang, Yi Li, Lang Liu, Zengliang Zang

**Affiliations:** ^1^College of Meteorology and Oceanography, National University of Defense Technology, Changsha, China; ^2^Key Laboratory of High Impact Weather (Special), China Meteorological Administration, Changsha, China

**Keywords:** random forest, prediction, meteorological factors, human health, pollutant

## Abstract

In recent years, the random forest model has been widely applied to analyze the relationships among air pollution, meteorological factors, and human health. To investigate the patterns and influencing factors of respiratory disease-related medical visits, this study utilized data on medical visits from urban areas of Tianjin, meteorological observations, and pollution data. First, the temporal variation characteristics of medical visits from 2013 to 2019 were analyzed. Subsequently, the random forest model was employed to identify the dominant influencing factors of respiratory disease-related medical visits and to construct a statistical forecasting model that relates these factors to the number of visits. Additionally, a predictive analysis of medical visits in Tianjin for the year 2019 was conducted. The results indicate the following: (1) From 2013 to 2019, the number of medical visits exhibited seasonal fluctuations, with a significant decline observed in 2017, which may be directly related to adjustments in hospital policies. (2) Among the meteorological factors, average temperature, relative humidity, precipitation, and ozone concentration significantly influenced the variation in medical visits, while wind speed, precipitation amount, and boundary layer height were of lesser importance. Furthermore, different linear relationships exist among the meteorological factors; specifically, meteorological factors show a negative correlation with pollutant elements, and there is a strong correlation among the pollutant factors. (3) When the number of medical visits ranged from 50 to 200, the predictions made by the random forest model closely matched the actual values, demonstrating strong predictive performance and the ability to effectively forecast daily variations in medical visits over extended periods, thus exhibiting good stability and generalization capability. (4) However, since the random forest model relies on a large amount of data for model validation, it has limitations in capturing extreme variations in medical visit numbers. Future research could address this issue by integrating different models to enhance predictive capabilities.

## 1 Introduction

Over the past century, fossil fuel combustion and unequal, unsustainable energy and land use have caused a global temperature increase of 1.1°C above pre-industrial levels (1–3). This rise has led to more frequent and intense extreme weather events, along with increasingly severe impacts on nature and people in all regions. Further increases in global temperature are expected to intensify these hazards. More severe heatwaves, heavier rainfall, and other extreme weather events pose greater risks to human health and ecosystems. Extreme heat has resulted in deaths in every region. As the planet continues to warm, the negative effects of climate change on food and water security will grow. When these risks combine with other challenges, such as epidemics and conflicts, the situation becomes even harder to manage. The focus on loss and damage highlights that the most vulnerable people and ecosystems experience the greatest losses and damages (4).

A growing body of research shows that climate change is creating widespread health risks globally. It is intensifying heatwaves, increasing wildfires, raising flood risks, and worsening droughts (5). These changes contribute to higher rates of heat-related mortality, pregnancy complications, and cardiovascular diseases. As with many climate-related issues, the risks and hazards are most severe in regions with the least capacity to adapt (6, 7).

Hospitals worldwide have faced significant challenges due to extreme weather (8). Many are increasingly unprepared to handle storms, high temperatures, and other climate-related events that are becoming more frequent. Floods claimed the lives of COVID-19 patients at a hospital in Mexico (9). Severe flooding affected hospitals in India. Hospitals on the West Coast struggled to maintain indoor air quality during wildfires. Hurricanes damaged the roof of a rural hospital in Louisiana (10–12).

In recent years, as living standards have improved, health has become an important focus of daily life (13–15). The desire for better health and longer lives has become increasingly urgent. Traditional weather forecasts are no longer sufficient to meet public needs, leading to a growing demand for more specific and professional forecasting. Because of the strong link between meteorological conditions and health, it is essential to conduct in-depth research on medical meteorology (16, 17). This includes developing meteorological indicator systems and prediction models tailored to local health needs. Medical meteorological forecasting aims to help individuals take preventive measures and assist medical institutions in preparing for disease prevention and control in a more targeted and timely manner (18).

The relationship between the meteorological environment and human health is complex. It often follows a nonlinear pattern and is influenced by additional non-environmental factors. In recent years, the promotion and application of random forest models have significantly advanced the understanding of this correlation (19). Random forest is an algorithm designed to control the overfitting tendency of decision trees. It achieves this through bagging (bootstrap aggregation), which involves sampling the training set with replacement. This method effectively handles collinearity and interactions among variables, including other independent variables and external factors. It also compensates for missing or incomplete inputs, making it a flexible tool for analyzing the impact of intervention measures on air quality time series (20–23).

The random forest model is particularly useful when the number of explanatory variables is large, the relationship between the response and explanatory variables is unclear, or the response variable does not conform to specific distribution requirements (24). While relatively few studies have applied random forest models for prediction, existing research highlights their strong fitting performance. In China, medical meteorological forecasting is still in its early stages. Advancements in science and technology, along with increasing environmental awareness, are expected to drive further research in this area. These developments will likely promote the growth of medical meteorological forecasting and its broader practical applications (25).

Currently, research on predicting patient numbers using machine learning models and examining the effects of climate conditions (e.g., temperature, humidity, pressure, wind, and pollutants) on respiratory diseases primarily focuses on average monthly data and short-term forecasts. Most studies rely on ARIMA, GAM, and GLM models (26). However, these methods often struggle to capture the complexity of non-linear relationships and interactions among multiple environmental factors (27, 28). In contrast, ARIMA excels at modeling time series with strong temporal patterns, but its capacity to handle nonlinear relationships is limited, making it more suitable for univariate or low-dimensional datasets. GAM provides a balance between nonlinear modeling and interpretability, but it requires careful selection of smoothing functions and struggles with temporal dependencies without extensions. In the study of urban heatstroke found that the evaluation and indicate that the random forest model stands out among all the compared models with its smallest MSE, RMSE, and *R*^2^ value closest to 1, which suggests that it has higher accuracy in predicting the number of heatstroke victims per day (29).Our study addresses these limitations by employing the Random Forest (RF) model, which is better equipped to handle these challenges. By doing so, we aim to improve predictive accuracy and deepen the understanding of how environmental variables influence respiratory health (30).

The random forest model offers several advantages over other statistical models and is particularly effective in fitting the nonlinear effects of meteorological factors (31–35). First, it accounts for interactions between variables and handles datasets with a large number of features efficiently. Second, by introducing double randomness—random sampling of training data and random selection of variable subsets—it enhances resistance to overfitting (36). Third, it is highly robust to missing values and outliers, reducing the influence of outliers by averaging the results of all regression trees. Fourth, it requires relatively few parameters for model construction. Finally, the model-building process inherently includes cross-validation, enhancing reliability (37). These features make the random forest model a powerful tool for analyzing complex environmental and health data.

Tianjin is situated in the northern temperate zone, on the east coast of the Eurasian continent in the mid-latitudes (38). Its climate is primarily influenced by the East Asian monsoon circulation, resulting in a warm temperate semi-humid monsoon climate. Proximity to Bohai Bay further amplifies the influence of the marine climate. As a major city in one of China’s three key economic regions along the eastern coast and one of the three major metropolitan areas in Northeast Asia, Tianjin plays a pivotal role in regional development (39, 40). It serves as a modern port city, a vital hub for sea-land transport in Northeast Asia’s urban corridors, and a key connection point for the “Belt and Road” initiative. Investigating the effects of meteorological changes and pollutant concentrations on respiratory diseases in Tianjin provides critical insights into the relationships between human activities, meteorological factors, and atmospheric environmental quality. Moreover, these findings have significant practical applications for environmental planning, urban development, pollution control, and public health management in the Beijing-Tianjin-Hebei region (41). Employing random forest models to forecast outpatient respiratory disease caseloads enables more effective public health interventions within urban environments. These projections facilitate crucial actions including the optimization of healthcare resource allocation, the dissemination of timely public health advisories, and the implementation of preventative measures designed to mitigate the impact of predicted increases in respiratory disease.

## 2 Methods and data

### 2.1 Data

The data used in this article include respiratory outpatient medical data, meteorological data, and pollutant data. Respiratory outpatient medical data includes the number of outpatient visits to the respiratory department, patient age and gender during the period from April 2013 to December 2019. Meteorological data comes from the Tianjin Meteorological Bureau, including observation data of meteorological elements such as temperature, relative humidity, wind speed, and precipitation. The hourly concentration monitoring data of pollutants (NO_2_, SO_2_, O_3_, PM_2.5_, PM_10_) comes from the National Urban Air Quality Real-time Publishing Platform.^1^

To ensure the accuracy of the research results, strict adherence was maintained to the “Ambient Air Quality Standards” (GB3095-2012). Quality control was applied to the original data from the monitoring stations, which involved removing missing values and outliers from the environmental variables. Ultimately, a total of 414,887 valid data points were selected, resulting in a data missing rate of 0.12%.

### 2.2 Random forest

Random forest is an ensemble learning method that combines multiple decision trees, where each tree is built using independently sampled random vectors from the original data (Bagging algorithm) (42). The algorithm optimizes node selection for each independent variable based on the residual sum of squares (RSS). At each node, it splits the data to minimize the residuals of the two resulting subsets, thereby enhancing predictive accuracy.

The training dataset for the random forest model included daily records of outpatient visits to the respiratory department from April 2013 to December 2018. The validation dataset, used for evaluating model performance, consisted of daily outpatient data from 2019. The model inputs included meteorological variables, pollutant variables and temporal variables (date_unix, day of the year, weekday, and hour of the day). The date_unix variable represents the number of seconds since January 1, 1970 (43). It was used to explain hourly mean concentrations of pollutant data. This approach allowed for the integration of temporal and meteorological factors, providing a robust framework for analyzing air quality data. The random forest model was implemented using the latest “rmweather” R package.

### 2.3 Feature importance analysis using the SHAP algorithm

SHAP (Shapley Additive Explanations) is a widely adopted method in interpretable machine learning, designed to explain the prediction outcomes of complex models. The SHAP framework assigns an importance weight to each feature, quantifying its contribution to the model’s predictions. This approach provides insights into how individual features influence model behavior, making it a valuable tool for understanding machine learning models (42).

Compared to other interpretable machine learning methods, SHAP offers several distinct advantages:

1. Consistency: SHAP ensures both global and local consistency in its explanations, which enhances the reliability and stability of the results.
2. Local Accuracy: SHAP provides feature importance at the individual sample level, enabling detailed explanations for single predictions.
3. Feature Importance Ranking: SHAP ranks features by their importance, helping users identify the most significant predictors and facilitating feature selection.

## 3 Results

### 3.1 Statistical description

#### 3.1.1 Change characteristics of the number of medical visits

**Figure 1:**
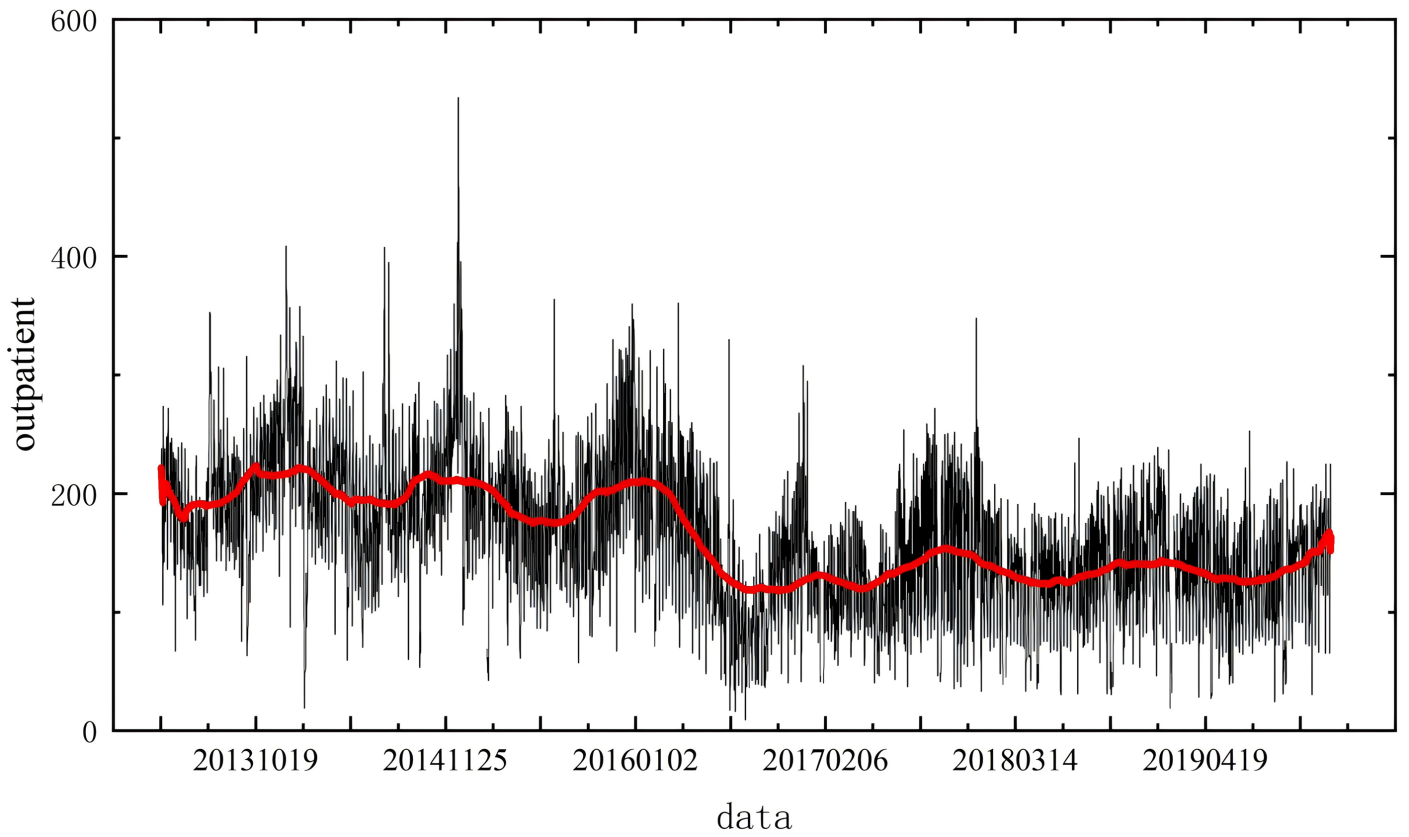
Daily average changes in the number of outpatient visits to the respiratory department.

The cumulative number of respiratory system consultations in Tianjin from April 2013 to December 2019 was 414,887. Figure 1 illustrates the time series of outpatient visits during this period, revealing a cyclical annual pattern. Each year, the number of visits typically peaked at the beginning and end of the year, with lower numbers in the middle months.

Daily outpatient visit data were collected for the 81-month period from April 2013 to December 2019. The average number of daily visits was 225.50, with a minimum of 9 and a maximum of 847 cases per day. The trend in daily visits, shown in Figure 1, exhibits significant fluctuations. Notably, there are two peak periods annually. However, a marked decline in the number of visits occurred from 2016 to 2017. Analysis suggests that this decline coincided with institutional changes at the hospital, including a reduction in doctors’ weekend consultation hours. These external factors substantially affected the number of patients and must be accounted for in the modeling process to minimize their impact on predictions.

**Figure 2:**
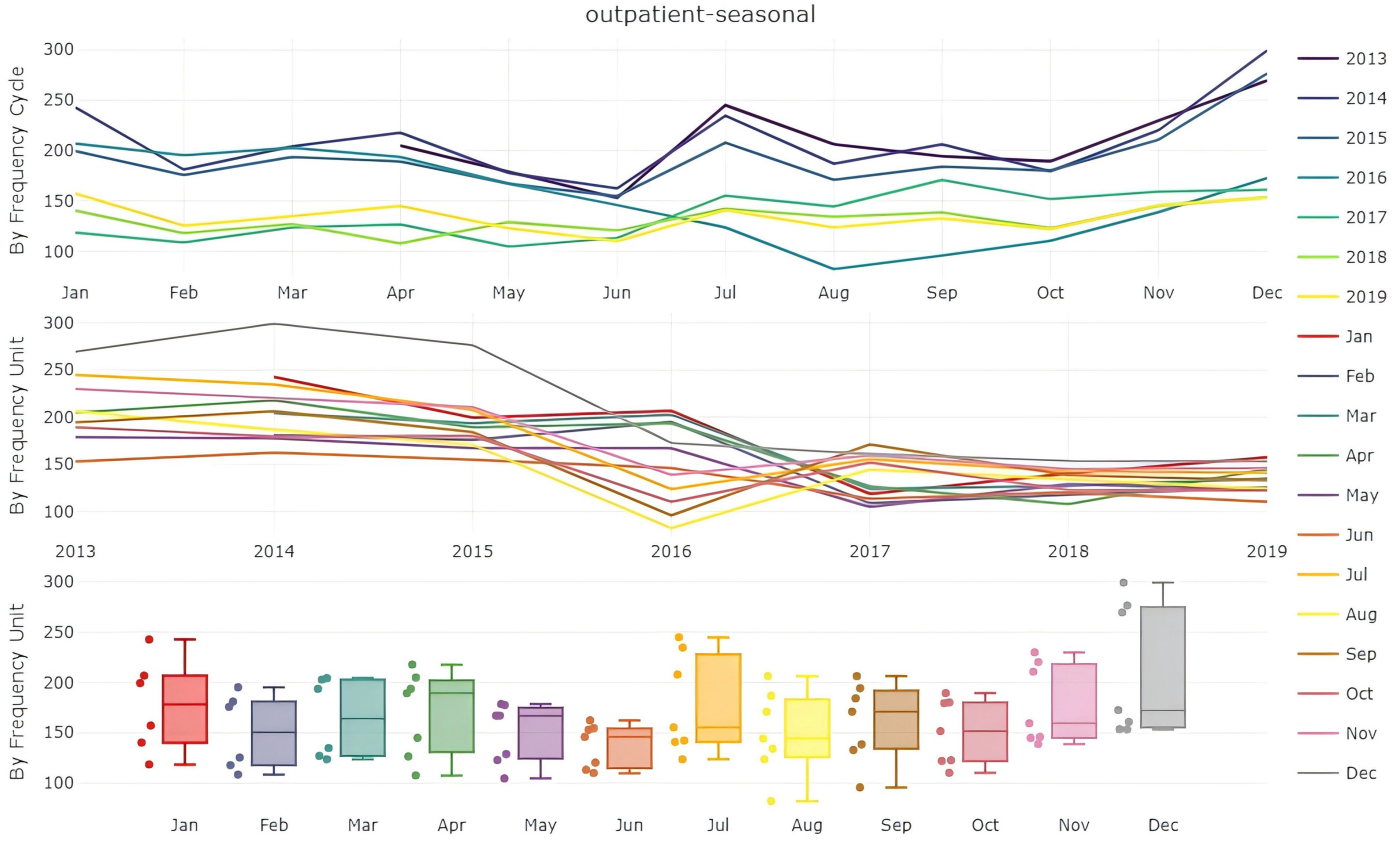
Analysis of the monthly average number of patients.

Seasonal analysis (Figure 2) highlights distinct patterns in outpatient visits. A minor peak occurs in July, while a sharp increase begins in October as temperatures drop during winter. This seasonal trend indicates a higher frequency of medical consultations in both summer and winter. Box plot analysis for each month further reveals significant variability in outpatient visits during July and the winter months, with both higher patient numbers and larger fluctuations observed.

The COVID-19 pandemic in early 2020 profoundly disrupted hospital outpatient services. As of July 2021, the monthly number of visits had not returned to pre-pandemic levels. February also consistently shows a decline in outpatient visits each year due to the Chinese Lunar New Year, during which fewer people seek medical care. Given the significant impact of the pandemic on outpatient numbers after January 2020, only data from 2013 to 2018 were used to develop the predictive model. Data from 2019 were subsequently employed for model validation.

### 3.2 Analysis of changes in meteorological elements and pollutant concentrations

The summary statistics for patient numbers, daily average meteorological factors, and pollutant concentrations from 2013 to 2018 are presented in Table 1. The data indicate that the average temperature, maximum temperature, and minimum temperature in Tianjin during this period were 289 K, 309.9 K, and 259.6 K, respectively. The average relative humidity was 44.70%, and the average wind speed was 4.28 m/s. Regarding air pollutants, the average concentrations of PM_10_ and PM_2.5_ were 110.15 and 68.45 mg/m^3^, respectively. The average concentrations of O₃, NO₂, SO₂, and CO were 57.30, 47.27, 25.11, and 1.36 mg/m^3^, respectively.

**Table 1:** Frequency distribution of main meteorological elements and air pollution in Tianjin from 2013 to 2018.

	Mean	Minimum	P25	Median	P75	Maximum
Wind_speed (m/s)	4.28	0.82	3.19	4.10	5,015	11.77
Temperature (K)	289	259.60	277.90	291.10	299.2	309.90
Relative-humidity (%)	44.70	6.09	29.58	43.70	57.91	94.25
Planetary-boundary-layer-height (m)	520.01	42.28	347.69	489.56	647.4	2048.84
Precipitation (mm)	18.54	0.73	5.93	13.55	28.39	72.62
Pm_10_ (μg/m^3^)	110.15	10.66	63.55	93.84	138.55	483.60
Pm_2.5_ (μg/m^3^)	68.45	6.86	34.30	55.15	87.36	383.98
O_3_ (μg/m^3^)	57.30	2.81	26.55	50.56	79.57	191.07
No_2_ (μg/m^3^)	47.27	9.14	31.20	43.08	59.62	176.20
So_2_ (μg/m^3^)	25.11	2.41	9.26	15.41	29.03	237.13
Co (mg/m^3^)	1.36	0.29	0.88	1.18	1.62	8.46

From 2013 to 2019, the average temperature in Tianjin was 289 K, with a maximum temperature of 309.90 K and a minimum temperature of 259.60 K. These data suggest that Tianjin experiences a relatively mild climate with pronounced seasonal variations. The average relative humidity was 44.702%, indicating a moderately dry environment that could influence public health and daily life. The average wind speed of 4.28 m/s reflects relatively stable wind conditions, which may play a role in the dispersion and dilution of air pollutants.

Tianjin’s air quality indicators reveal significant pollution levels. The average PM_10_ concentration was 110.15 μg/m^3^, and the average PM_2.5_ concentration was 68.45 μg/m^3^, both of which exceed the safety thresholds set by many countries. These particulate matters are known to irritate the respiratory system. PM10 particles, when inhaled, can cause conditions such as asthma and chronic bronchitis. PM2.5, due to its smaller size, can penetrate deeper into the alveoli and bloodstream, potentially impacting the cardiovascular system.

The average concentrations of other air pollutants further highlight the severity of Tianjin’s air quality challenges. Ozone (O₃) averaged 57.30 μg/m^3^, nitrogen dioxide (NO₂) 47.27 μg/m^3^, sulfur dioxide (SO₂) 25.19 μg/m^3^, and carbon monoxide (CO) 1.39 μg/m^3^. These gases, particularly in industrial and high-traffic areas, contribute to poor air quality. Elevated levels of ozone and nitrogen oxides are known to irritate the respiratory tract and may trigger asthma attacks or exacerbate lung diseases.

Tianjin’s geographical location and rapid industrialization have created favorable conditions for air pollution. As a northern coastal city, Tianjin has a dense industrial infrastructure, including numerous manufacturing plants, petrochemical enterprises, and energy industries, which release substantial pollutants during production. Additionally, the increasing use of motor vehicles has made exhaust emissions a major source of urban air pollution. Growing traffic volumes have further deteriorated air quality, particularly in the city center.

In addition, as urbanization progresses, the green coverage rate remains relatively low, and the urban heat island effect is becoming more prominent. This effect leads to higher temperatures, which in turn promote the formation of ozone. Elevated temperatures facilitate chemical reactions, resulting in increased ozone concentrations. At the same time, high humidity conditions may cause pollutants to accumulate and form haze. This phenomenon is particularly noticeable during the autumn and winter months, often leading to reduced visibility and, in some cases, meteorological disasters.

Extended exposure to such environmental conditions significantly increases health risks. Studies have shown that poor air quality is directly linked to the incidence of chronic respiratory diseases, cardiovascular diseases, and lung cancer. Children and the older adult are especially vulnerable to these pollutants, often experiencing allergic reactions or acute health issues. Pregnant women living in highly polluted environments may face risks to fetal development, including complications such as low birth weight. In recent years, Tianjin has faced a severe air pollution problem, which is closely related to its climate, geographical features, and human activities. In rapidly developing cities, it is essential for the government to implement effective policies to improve air quality. The public’s health and quality of life must be prioritized. Only through raising environmental awareness and strengthening pollution control measures can a healthier living environment be created for citizens.

### 3.3 Correlation between main meteorological factors and daily emergency room visits for respiratory diseases in Tianjin from 2013 to 2019

**Figure 3:**
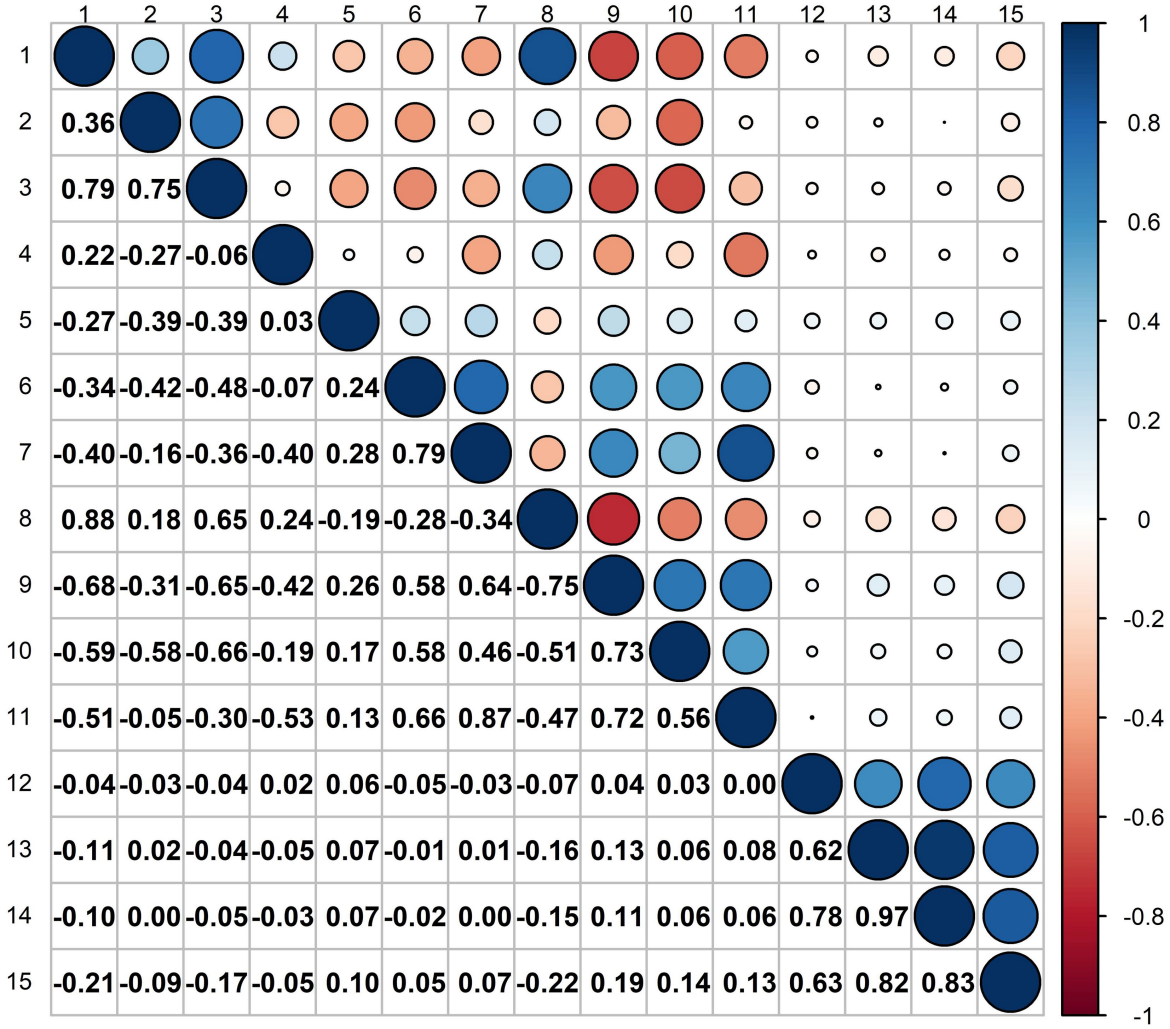
Correlation analysis of various factors on the number of patients. 1. Average temperature, 2. Relative humidity, 3. Precipitation, 4. Wind speed, 5. Boundary layer height, 6. Pm_10_, 7. Pm_2.5_, 8. o_3_, 9. No_2_, 10. So_2_, 11. co, 12. Clinic hours, 13. Number of doctors, 14. Medical resources (the product of clinic hours and number of doctors), 15. Number of patients; the value range is between −1 and 1, where −1 indicates a complete negative correlation, 1 indicates a complete positive correlation, and 0 indicates no linear relationship; the area of the circle indicates the absolute value of the correlation coefficient, and the color depth indicates the strength of the correlation.

Figure 3 presents the Spearman’s correlation between key meteorological elements and the number of daily emergency department visits for respiratory diseases in Tianjin from 2013 to 2019. Temperature, relative humidity, precipitation, and ozone are all negatively correlated with the number of outpatient visits for respiratory diseases to varying degrees. Among these, the correlation between average temperature and ozone is the strongest, followed by the effect of precipitation.

Overall, the influence of temperature is particularly significant. Current research indicates that among various meteorological factors, temperature and humidity are closely related to the incidence of respiratory diseases. A recent study conducted by scholars from Finland and the United Kingdom found that when room temperature ranges from 18 to 26°C and relative humidity is between 17 and 40%, increasing humidity helps alleviate dryness in the nose, reduces nasal congestion, and relieves symptoms of a dry throat. Conversely, an increase in room temperature can exacerbate dry throat symptoms. This suggests that there is an optimal range for the effects of temperature and humidity on the respiratory system. Extremes in either temperature or humidity, whether too high or too low, can have detrimental effects on respiratory health. This implies that the combined effects of temperature and humidity on the respiratory system are nonlinear. Furthermore, short-term exposure to high concentrations of ozone can lead to respiratory irritation, including symptoms such as sore throat, cough, and wheezing. Prolonged exposure to ozone may result in reduced lung function and an increased risk of chronic respiratory diseases. Additionally, ozone exposure can cause lung tissue inflammation, impair alveolar function, and reduce the efficiency of oxygen exchange. Therefore, it is crucial to protect the respiratory system by minimizing exposure to ozone.

(1) The impact of individual meteorological factors on the number of patient visits: In this study, we calculated the Spearman correlation coefficients between 14 factors and the number of patients to assess the strength of their relationships. The coefficients for the number of doctors and the operating time of medical resources were 0.82 and 0.83, respectively, indicating a strong positive impact on patient visits. As the number of doctors increases or medical resources expand, patients are more likely to seek treatment due to reduced waiting times and improved satisfaction. The correlation between time and the number of patients was 0.63, suggesting that longer time provide more opportunities for patients to seek care. In contrast, environmental factors, such as air pollution and climate conditions, exhibited relatively low correlations with patient visits, most of which were negative. For instance, the Spearman coefficients for PM_10_ and PM_2.5_ were 0.05 and 0.07, indicating minimal effects. Negative correlations were also observed for relative humidity (−0.09), precipitation (−0.17), wind speed (−0.05), and boundary layer height (0.10), suggesting that adverse weather conditions may discourage hospital visits. Higher humidity and precipitation could deter individuals from going out, while strong winds might negatively affect physical health, leading people to stay home. Furthermore, the coefficients for pollutants such as O_3_, NO_2_, SO_2_, and CO were − 0.22, 0.19, 0.14, and 0.13, respectively, with the negative correlation for O_3_ indicating that higher ozone levels may reduce the likelihood of seeking medical treatment, particularly for individuals with pre-existing respiratory conditions. Additionally, the correlation coefficient for average temperature and patient visits was −0.21, revealing a significant negative relationship. This suggests that extreme temperatures, both high and low, may discourage individuals from seeking medical attention. Hot weather may prompt people to remain indoors, thereby reducing healthcare visits, while sudden temperature fluctuations could exacerbate seasonal health conditions, leading to temporary increases in patient numbers. Overall, extreme temperature conditions tend to negatively impact medical-seeking behavior.
(2) The linear relationships among meteorological factors: The correlation coefficient is a statistic used to measure the strength and direction of the linear relationship between two variables. Based on the correlation coefficients shown in Figure 3, it can be observed that the lighter the color of the number, the weaker the linear relationship between the two meteorological factors. Among these factors, the boundary layer height has a relatively weak correlation with other factors (correlation coefficient < 0.5). The average temperature and precipitation have a more pronounced effect on pollutant factors compared to other meteorological variables. Additionally, there is a strong correlation among the pollutant factors.

The combined influence of environmental factors and medical resources provides an important context for changes in patient numbers. A complex interaction exists between the availability of medical resources, doctors’ work schedules, and the impact of environmental factors on public health. Environmental risks may drive people to seek medical services, while the availability of healthcare resources determines their access to care. Thus, enhancing the allocation of medical resources and improving environmental quality are critical for promoting better health outcomes and healthcare utilization. Together, these factors interact through various mechanisms, influencing individuals’ decisions to seek medical attention and, ultimately, contributing to changes in patient numbers.

### 3.4 Establishment of respiratory disease prediction model

**Figure 4:**
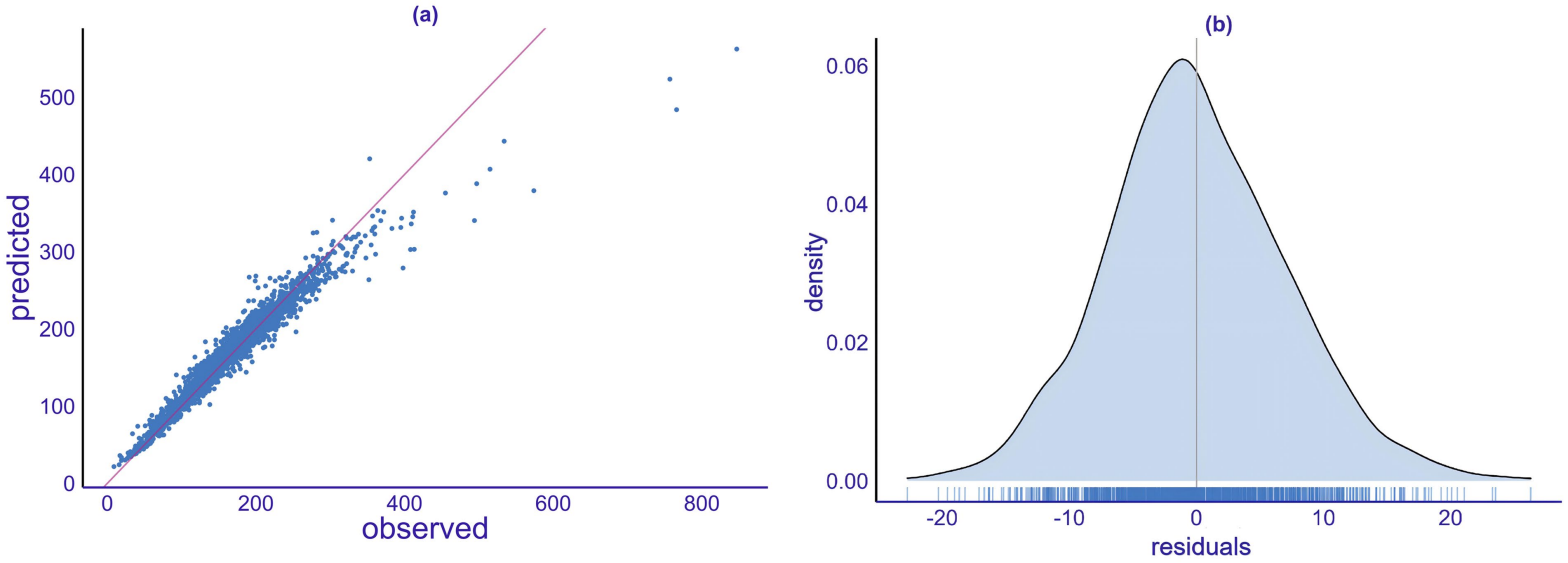
Model fitting analysis. **(a)** Quantile-quantile plot. **(b)** Density plot of the model’s predicted residuals.

A prediction model was developed using daily emergency department visit data for respiratory diseases in Tianjin from 2013 to 2018. The model accounts for various factors, including long-term trends, weekly and holiday effects, as well as meteorological variables such as average temperature, relative humidity, average wind speed, boundary layer height, and precipitation. Additionally, air pollution levels (PM_10_, O_3_, SO_2_, NO_2_, etc.) were incorporated to assess their combined impact. Figure 4 presents a regression scatter plot of the model fitting. The data points are evenly distributed around the solid line, indicating that the random forest model explains more than 80% of the variation in patient numbers, demonstrating its strong predictive performance. The model exhibits a small error between predicted and observed values, further confirming its reliability and providing a solid foundation for future research.

The goodness-of-fit analysis evaluates the existing predictive model. In this study, we employed linear regression to test the model (Figure 4a). By inputting the original data into the model, we obtained the fitted values and compared these results with the actual observed values. The figure indicates a close linear relationship between the predicted values and the actual values. Most data points are concentrated near the regression line, suggesting a good fit between the predicted and actual values. Additionally, the slope of the regression line is close to 1, indicating an accurate proportional relationship between the predicted and actual values. However, there are a few outliers, which may result from not considering all influencing meteorological factors or from random errors in the data.

The residual plot can be used to assess whether the model’s residuals are consistent with random errors. Figure 4b presents the density plot of the model’s predicted residuals, which helps evaluate the model’s fit. The horizontal axis represents the magnitude of the residuals, while the vertical axis indicates the corresponding density of the residuals. The height of the density reflects the proportion of the residuals and allows for a visual assessment of their distribution, providing a preliminary judgment of the model’s fit. The results in Figure 4B show that the distribution of the model’s residuals follows a unimodal, bell-shaped curve, consistent with a normal distribution.

### 3.5 Establishm model interpretability analysis

#### 3.5.1 SHAP analysis

**Figure 5:**
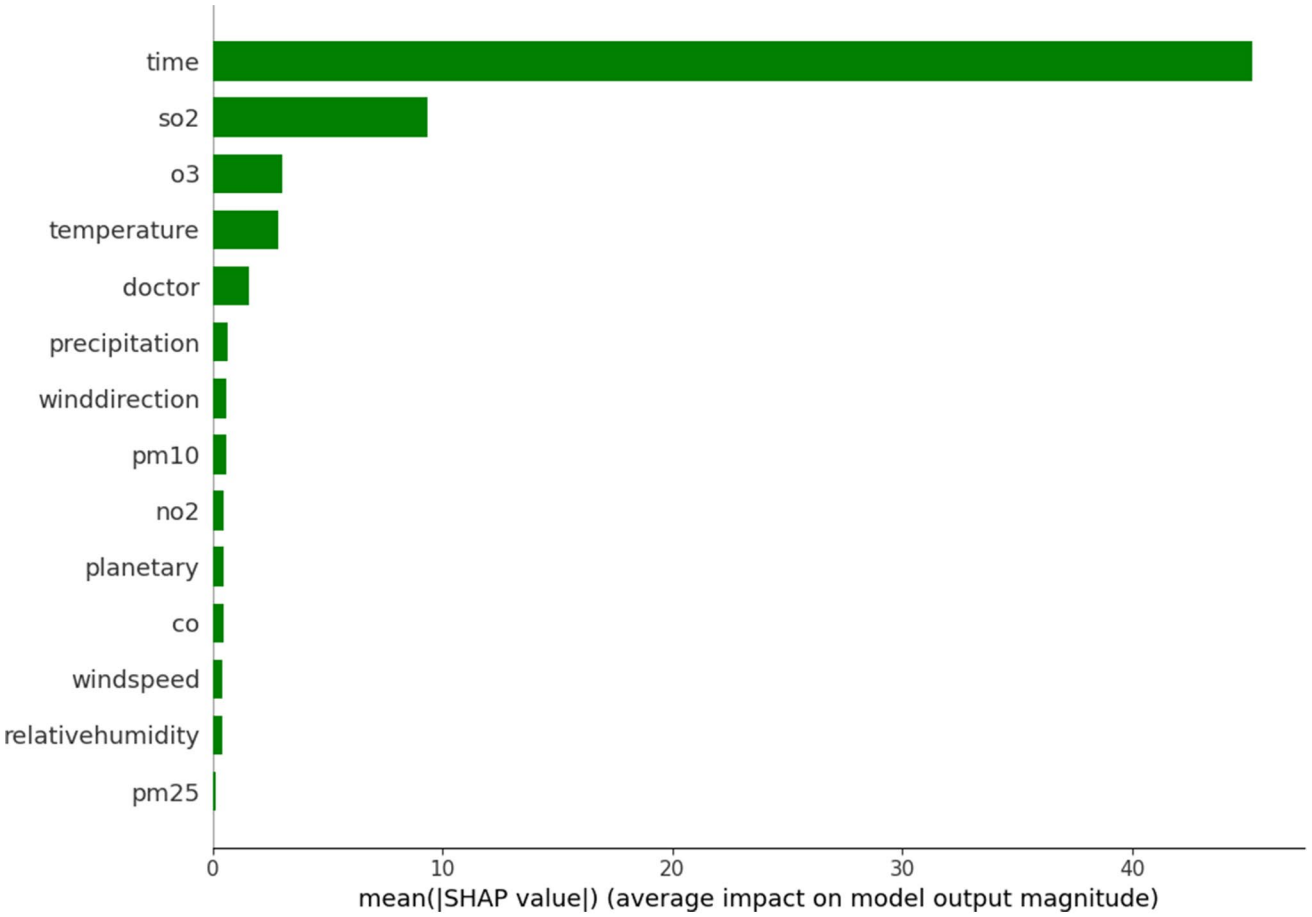
Importance of single factor variables.

**Figure 6:**
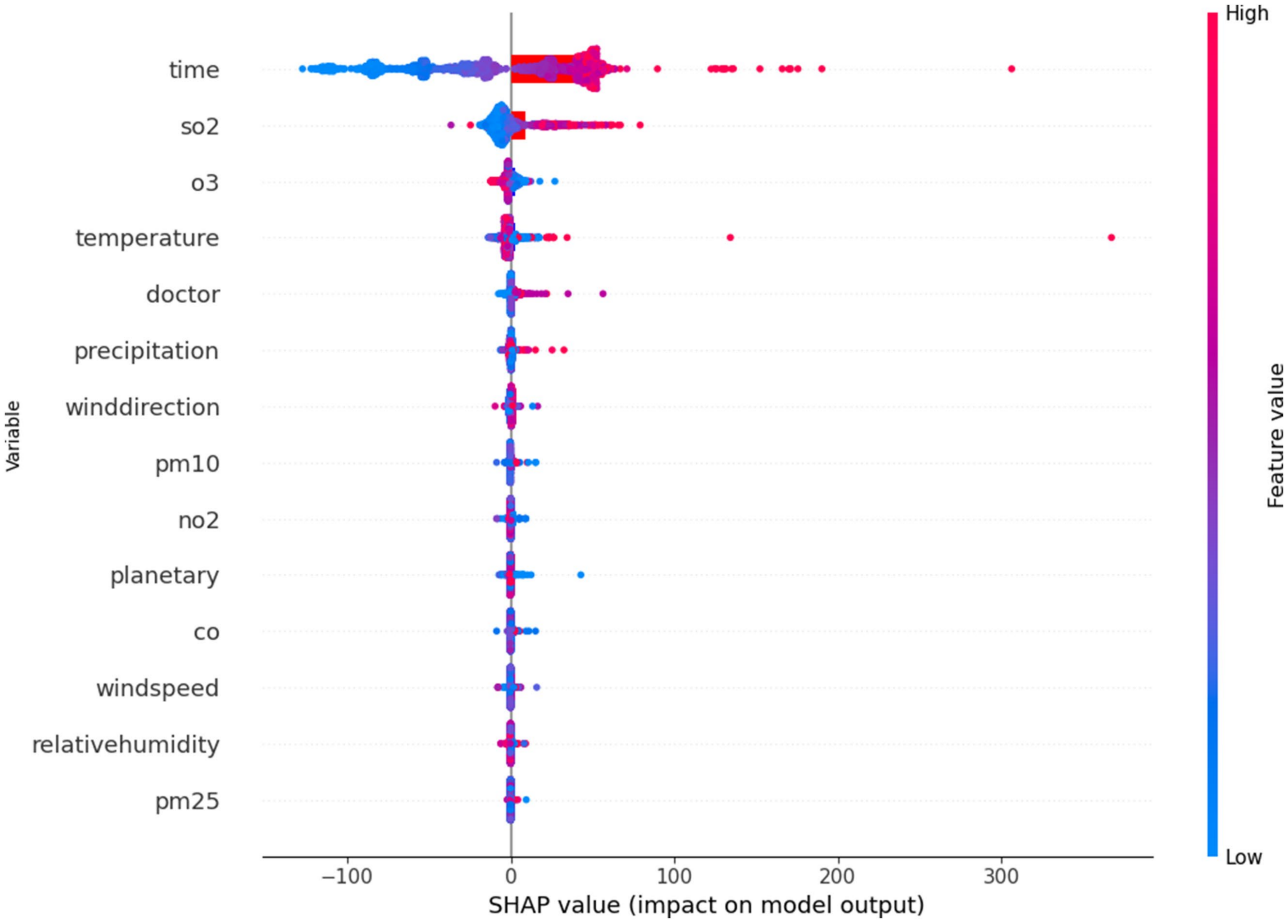
SHAP analysis.

To evaluate the importance of different meteorological variables, the SHAP model was applied. Figure 5 illustrates the importance ranking of individual variables, while Figure 6 shows the distribution of SHAP values derived during the construction of the random forest model.

Variable importance plays a critical role in selecting predictors for the random forest model. In this study, meteorological data were used as pre-selected influencing factors. Redundant variables with weak correlations were eliminated based on their importance, thereby streamlining the prediction model and enhancing its operational efficiency. For the case of Tianjin, the pre-selected influencing factors were evaluated, as shown in Figure 5. This ranking indicates that the higher the influence of a factor, the greater its importance. Specifically, sulfur dioxide (SO_2_), ozone (O_3_), and temperature were identified as significantly more important than other meteorological factors. The findings suggest that the number of patients in Tianjin is most sensitive to variations in SO_2_, O_3_, and temperature.

When predicting the number of patients, the ranking of variables provides valuable insights into the influence of different factors. Among these, sulfur dioxide (SO_2_) and ozone (O_3_) ranked first and second, respectively, highlighting their significant impact on the number of patients. Sulfur dioxide, a major air pollutant, is strongly linked to respiratory diseases. Elevated concentrations of SO_2_ can irritate the airways, leading to symptoms such as coughing and wheezing, which may increase the demand for medical treatment. Ozone, a byproduct of chemical reactions between pollutants in the presence of sunlight, is more prevalent during the summer months. High ozone concentrations also have substantial negative effects on public health, with many individuals experiencing difficulty breathing, further contributing to an increase in medical visits. Thus, the high importance of these two pollutants can be understood as a direct threat to public health.

Temperature ranks closely behind other variables in its influence on the number of medical visits. Changes in temperature can affect the immune system and overall physiological condition. Cold weather often leads to an increase in respiratory infections, while hot climates may cause heat-related illnesses such as heat stroke. Consequently, temperature changes are a key factor influencing both people’s health and their medical behavior.

The variable “doctor” also emerged as an important factor. The number of doctors and their distribution significantly impact the frequency of medical visits. When there are fewer doctors in a region, patients may delay seeking treatment due to difficulties in accessing healthcare. Even when symptoms become severe, individuals may wait until the condition worsens before seeking medical attention. Thus, the availability of medical professionals directly influences patients’ willingness and ability to seek care.

Precipitation is another relevant factor. Heavy rainfall often leads people to stay indoors, reducing outdoor activities, which in turn can impact the spread of respiratory diseases. However, in cases of extreme weather, such as heavy storms, transportation disruptions may affect people’s ability to access medical services. Additionally, precipitation can influence the transmission of certain infectious diseases. For example, during the rainy season, increased mosquito breeding can lead to outbreaks of diseases like dengue fever, thereby driving an increase in medical visits.

Particulate matter and gaseous pollutants, such as PM_10_ and NO_2_, also rank highly in their importance. These pollutants have well-established negative effects on both the respiratory and cardiovascular systems. PM_10_ particles can penetrate the lungs and cause a variety of health issues, while nitrogen dioxide (NO_2_) often exacerbates respiratory conditions such as asthma when combined with other pollutants. This highlights the strong connection between air quality and public health.

As a professional environmental parameter, the planetary boundary layer appears in the variable importance ranking, highlighting its impact on local air quality. The height of the planetary boundary layer influences the diffusion of pollutants. A lower boundary layer results in higher pollutant concentrations, which degrade air quality and increase the number of medical visits. In contrast, a higher boundary layer aids in the dilution of pollutants, reducing their harmful effects on public health.

Wind speed and relative humidity are also relevant factors. High wind speed facilitates the dispersion of pollutants, reducing their concentration in a given area, which can alleviate pollution-related health issues. Relative humidity interacts with respiratory health, as a high humidity environment may promote the growth of mold and bacteria, leading to an increased number of visits for allergies or respiratory conditions.

Although fine particulate matter (PM_2.5_) ranks lower in the importance ranking, its health impacts should not be underestimated. PM_2.5_ can penetrate deep into the alveoli and even enter the bloodstream, causing significant damage to the heart and lungs. Despite its lower rank, its influence on the number of medical visits may vary across different regions and time periods.

Analyzing the importance ranking of these variables provides valuable insights into the complex relationship between environmental factors and public health. This model not only offers a scientific basis for health policy development but also provides essential information for managing and preventing medical needs. Factors such as pollutant levels, temperature, and the availability of medical professionals interact to shape individuals’ health status, which, in turn, influences changes in the number of medical visits. This underscores the importance of considering multiple environmental and social factors in public health management.

SHAP (SHapley Additive exPlanations) values quantify the impact of a specific feature value by comparing it to the prediction made when the feature assumes a baseline value.

Variables with high SHAP values are considered important, while meteorological variables with SHAP values close to zero are of lesser importance. As illustrated in the figure, the doctor’s sitting time contributes significantly to changes in the number of patients. Among the meteorological variables, temperature is shown to have a major impact on patient numbers. Additionally, sulfur dioxide and ozone, as pollutants, demonstrate a substantial contribution, highlighting the importance of addressing ozone pollution and acid rain in the context of respiratory diseases. Although rainfall is less important, it is associated with low SHAP values, which aligns with the concept of wet deposition of aerosols.

#### 3.5.2 Partial dependence analysis

Partial dependence plots are a useful tool for visualizing how specific features affect model predictions. Like permutation importance, partial dependence plots are computed after model fitting and applied to real, unmodified data. By examining the relationship between variations in environmental factors and hospital visits, the results suggest that the relationship between pollutant concentrations and health outcomes is complex. These findings highlight a strong connection between air quality and public health. For example, nitrogen dioxide (NO₂) concentrations exhibit a dual effect. At low concentrations (0–50 μg/m^3^), NO₂ is negatively correlated with the number of hospital visits, suggesting that improved air quality in this range may reduce respiratory disease incidences. However, as concentrations increase to 50–125 μg/m^3^, a positive correlation emerges, indicating that higher pollution levels are linked to a greater demand for medical treatment. This shift may be due to the heightened health risks associated with higher pollutant concentrations.

Similarly, ozone (O₃) demonstrates a comparable dual effect. In the range of 0–50 μg/m^3^, O₃ is negatively correlated with the number of patients, indicating better public health at lower pollution levels. However, as O₃ concentrations rise to 125–150 μg/m^3^, a positive correlation emerges, which may be due to the severe health risks posed by high ozone concentrations in the air. The negative correlation observed with relative humidity suggests that high humidity causes physical discomfort, particularly for individuals with respiratory diseases.

Regarding particulate matter, both PM_10_ and PM_2.5_ show a clear positive correlation with hospital visits. Increases in PM_10_ concentrations correlate with higher patient numbers, underscoring the adverse effects of fine particulate matter on public health. PM_2.5_, in particular, demonstrates the closest relationship with patient visits within the concentration range of 100–125 μg/m^3^. These particles can penetrate deep into the lungs, enter the bloodstream, and impair cardiopulmonary function. A similar positive correlation was observed for sulfur dioxide (SO₂), further reinforcing the detrimental health effects of airborne pollutants.

The boundary layer height also revealed a significant negative correlation with hospital visits within the range of 0–500 meters. Lower boundary layer heights may trap pollutants near the ground, worsening air quality and increasing respiratory disease-related visits. Precipitation generally shows a negative correlation with patient numbers at 0–50 mm, but turns positive at higher precipitation levels (50–70 mm). Precipitation helps reduce airborne pollutants, leading to fewer health issues. Additionally, complex interactions between increased humidity and changes in precipitation patterns contribute to public health outcomes.

**Figure 7:**
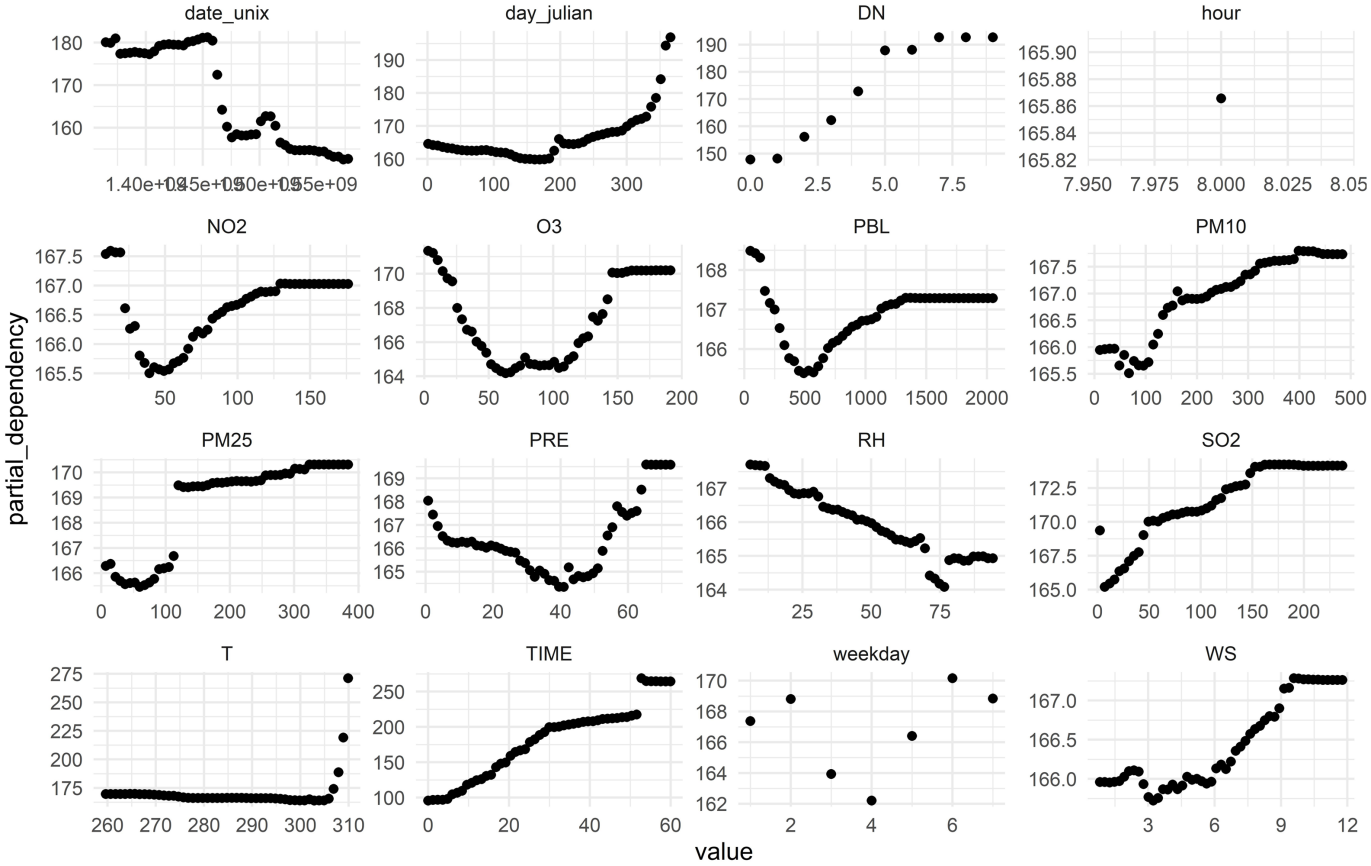
Partial dependence analysis of each factor on the number of patients.

Temperature also exhibits a clear positive correlation with hospital visits. High temperatures often exacerbate respiratory diseases, and hot weather increases ozone levels, further raising the demand for medical treatment. Wind speed has a more nuanced effect, showing a negative correlation at low wind speeds (0–5 m/s) and a positive correlation at higher wind speeds (5–9 m/s). This likely reflects the various mechanisms involved in wind-driven pollutant dispersion. At lower wind speeds, pollutants can accumulate in a specific area, negatively impacting public health. However, at higher wind speeds, pollutants may disperse or be diluted, improving air quality and reducing medical treatment needs (Figure 7).

### 3.6 Research on prediction and forecast of respiratory diseases

**Figure 8:**
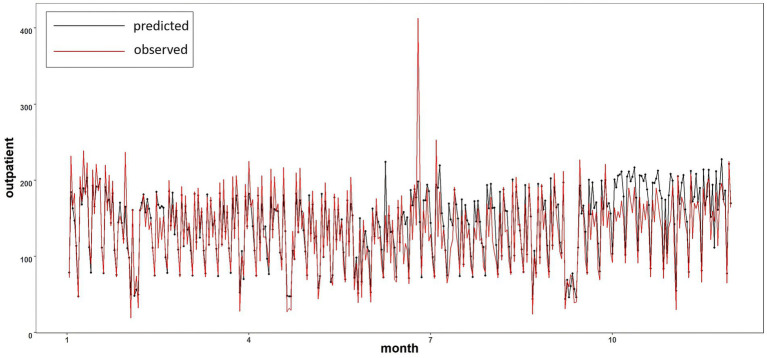
Comparison of predicted value and actual value in 2019.

To assess the prediction accuracy of the random forest model for ozone (O_3_) in estimating the number of daily emergency patients in Tianjin, the model was tested using data from 2013 to 2018 as the training set. The model incorporated 13 influencing factors, including average temperature, relative humidity, precipitation, wind speed, PM_10_, PM_2.5_, O_3_, NO_2_, SO_2_, CO, sitting time, number of doctors, and medical resources, to predict the daily number of respiratory patients for 2019. The predicted values were compared with the actual observed values (Figure 8). Figure 8 illustrates the distribution of the observed and predicted values for the number of respiratory patients in Tianjin in 2019. The high degree of consistency between the two suggests that the model performs well in predicting daily fluctuations in patient numbers. The strong goodness of fit indicates that the random forest model is effective for forecasting long-term daily changes in the number of respiratory patients in Tianjin.

## 4 Conclusion

(1) This study analyzed the temporal and seasonal variations in the number of outpatient visits for respiratory conditions in Tianjin from 2013 to 2019. The number of visits exhibited fluctuations with the seasons, with significantly higher numbers in summer and winter compared to spring and autumn. A notable decline occurred in 2017, which may be directly related to adjustments in hospital policies. Considering the trends in meteorological factors and pollutants, there is a clear overlap between the peaks in outpatient visits and temperature and precipitation during the summer. This suggests that the increase in outpatient visits may be associated with meteorological factors.
(2) The changes in outpatient visits show a nonlinear relationship with both meteorological and pollutant factors. Among these, average temperature, relative humidity, precipitation, and ozone have a strong correlation with the number of visits. The correlation between boundary layer height and other factors is weaker. The impact of average temperature and precipitation on pollutant factors is more pronounced compared to other meteorological elements. Additionally, there is a strong correlation among the pollutant factors. In the analysis of the importance of meteorological factors, it was found that sulfur dioxide, ozone, average temperature, and precipitation significantly influence the model’s predictions of outpatient visit numbers, while wind speed, precipitation, and boundary layer height have a smaller effect.
(3) When the number of outpatient visits is between 50 and 200, the random forest model demonstrates a high goodness of fit between predicted and actual values, indicating good predictive performance. This model effectively predicts the long-term daily variations in outpatient visits. Future work should focus on further optimizing model parameter selection and improving the model’s temporal resolution to achieve more accurate predictions.
(4) Although the random forest model effectively predicts long-term daily variations in outpatient visits, it lacks the ability to capture extreme values. This limitation arises because the random forest model relies heavily on large datasets for modeling and validation; insufficient sample sizes for extreme value data directly affect the model’s predictive accuracy. Therefore, to enhance the prediction of outpatient visits for respiratory conditions, future research could combine different models to improve predictive capabilities. Additionally, integrating this method with traditional mechanistic models, such as ARIMA, may yield more precise predictions while reducing time costs.

## Data Availability

The raw data supporting the conclusions of this article will be made available by the authors, without undue reservation.

## Footnotes

^1^ http://106.37.208.233:20035/
